# Why Do Some Users Become Enticed to Cheating in Competitive Online Games? An Empirical Study of Cheating Focused on Competitive Motivation, Self-Esteem, and Aggression

**DOI:** 10.3389/fpsyg.2021.768825

**Published:** 2021-11-29

**Authors:** Sung Je Lee, Eui Jun Jeong, Dae Young Lee, Gyoung Mo Kim

**Affiliations:** Department of Digital Culture and Contents, Konkuk University, Seoul, South Korea

**Keywords:** cheating, online games, self-esteem, aggression, competitive motivation

## Abstract

Cheating, the act of winning in a competition based on unfair advantages over one’s opponents, often occurs in online games (e.g., illegal money exchange, account hacking, and exploiting a bug). With the recent flourishing of competitive tournament games online, such as League of Legends (LoL) and Overwatch, cheating has emerged as a serious problem since it not only promotes the de-socialization of gamers but also adversely affects game brands. However, there has little research on this issue in studies on competitive online games. Focused on three psychological factors (i.e., competitive motivation, self-esteem, and aggression), which has been reported to be primarily related to cheating in sports, this paper presents a study that empirically examined the associations between the factors and cheating in competitive online game environments. From survey data of 329 LoL gamers in South Korea, a structural equation model was analyzed. The results showed that gamers with a high degree of competitive motivation are more inclined to cheat in the game. Aggression increased cheating behavior and had a significant relationship with competitive motivation. Self-esteem decreased the degree of cheating but did not affect competitive motivation. Notably, gaming time negatively influenced cheating. The practical implications of these study results were discussed.

## Introduction

With the recent developments in the e-sports industry, competitive online games, such as League of Legends (LoL), Overwatch, and Valorant, have won the favor of the public. These games are played by a selection of winners of fierce tournaments. All these games offer rewards, such as victory points, emblems, badges, and special items (or character skins), to the winners. As such, online games that highlight competition and rewards have leaped into prominence. However, cheating to gain unfair advantages over other players has emerged as a serious problem.

Cheating, in general, refers to actions designed to obtain personal gains with the use of various dishonest methods, such as deception, fraud, and violation of rules (Kräkel, 2007; Schermer, 2008). In online games, cheating means the act of achieving a task and winning a competition based on unfair advantages over one’s opponents (Consalvo, 2009, p. 87).

Cheating in the game appears in the form of obtaining virtual items through the illegal exchange of money, elo-boosting (a person’s participation in a competition on behalf of another person), account hacking, denying service to one’s peer players, glitching (exploiting a bug or loophole), exploiting another player’s misplaced trust, modifying the client infrastructure, etc (Yan and Randell, 2009). In addition, some players who want to more easily commit cheating with illegal programs, such as “map-hack,” “wall-hack,” “auto-targeting,” or Web sites and forums that share related information (Hamlen and Gage, 2011).

Cheating in online games could hinder normal and fair competition by allowing certain gamers to have significant benefits or conveniences that are not available in a normal way. For example, in South Korea, games like LoL have suffered decreases in their number of players and in their PC café market share due to rampant cheating. It was reported that LoL suffered great damage due to an illegal program called “Helper” (Lee, 2016). Following the accelerated deterioration of the game’s reputation due to cheating, the South Korean representative of LoL developer Riot posted a written apology on the player community portal and carried out measures to prevent relevant damage, such as developing and introducing cheating detection solutions (League of Legends, 2016). These facts indicate that the prevalence of cheating in online games may lead to distrust of the ability of the game company to operate the game or may result in economic losses. Thus, game companies have come to regard cheating as one of the most serious problems in the market and have taken various measures to solve it, such as increasing the number of their employees dedicated to detecting illegal programs and securing detection solutions.

As cheating in competitive online games shows a tendency to spread to the community through victimization and observation (Kim and Tsvetkova, 2020), efforts are urgently needed to prevent damage. In a related move, a study in South Korea revealed that when match fixing is prevalent on Battle-net, the official server of Starcrafts, the players who no longer trust the competitor matching function of the official server move to private servers, such as PGTour or Fish-Server (Lim, 2008). In a similar vein, another study reported the “gold farming” phenomenon, in which illegal programs are used to abnormally acquire virtual goods, which results in damage (Seo et al., 2012).

Despite these efforts, however, there still has been a poor academic understanding of cheating in online games. Although attempts have been made to analyze the qualitative characteristics of cheating or to identify the psychological factors affecting such misbehavior (Yan and Randell, 2009; Wu and Chen, 2013, 2018; Chen and Wu, 2015), many studies on cheating in online games focused on technical approaches to detecting cheating in games (Botvich et al., 2010; Alayed et al., 2013; Kang et al., 2013). In particular, considering the social and industrial concerns on cheating in online games, more studies are urgently needed.

In this regard, this paper explored how psychological factors affect cheating in competitive online gaming environments. Specifically, this study focused on three primary factors (i.e., competitive motivation, self-esteem, and aggression) based on previous studies on sports and tested a structural equation model to investigate the factors’ associations with cheating in online games from survey data of 329 LoL players in South Korea.

## Literature Review

### Moral Reasoning and Illegitimated Regression

Moral reasoning refers to the process of deliberating over reason in making or revising moral decisions (Stanley et al., 2018). It may occur in exceptional situations at a different level from what is usually experienced in daily life (Bredemeier and Shields, 1986a; Shields and Bredemeier, 2008). In such situations, an individual faces a moral dilemma and exhibits a lower level of moral reasoning than normal. For example, in the prevailing moral atmosphere of the negative situation when exposed to considerable violence, individuals may exhibit lower levels of moral reasoning than normal (Kohlberg et al., 1971; Posada and Wainryb, 2008).

Sports and games take place in a special situation that is distinct from everyday situations. They form a play space through a magic circle that serves as a boundary that separates the virtual world and the rest of the world. The magic circle refers to a physical and normative indicator that leads a specific time, space, and context into a playful situation (Juul, 2011). Thus, the space-time of sports has an exceptionality that is distinct from the everyday real world through the indicators of whistles and flags (Schmitz, 1979; Shields and Bredemeier, 2008). When a player in uniform enters the playing field, it means one is entering an exceptional space of play, and the whistle signals the beginning of an exceptional moment.

Magic Circle’s indicators confirm that sports and games will be conducted within a set range and rules. For example, sports, such as boxing, are not as unrestricted as street fights and are only valid in prescribed time and space. In addition, all actions are bound by rules recognized by the authorities through consensus (judgment, scoring system, method of surrender, etc.) and will be sanctioned if they are not performed. And this means that the space–time of the sport formed by the Magic Circle has a non-oneness and exceptionality.

Players in sports and games pursue extraordinary goals and values through observance of the play rules that constitute an exceptional situation. The action of putting a ball into the goal post acquires meaning through observance of play indicators and rules, but in everyday life, it does not mean more than the action itself. As a result, sports and games are guaranteed exceptionality characteristics through the magic circles and rules that determine the playful time and space. Sports also justify the desire to fight and the outburst of aggression through formal rules and norms. Considering the levels of moral reasoning in exceptional situations, an individual could temporarily show immature moral reasoning, such as egocentrism while participating in sports or games, and this is called “legitimated regression” (Bredemeier and Shields, 1986a; Shields and Bredemeier, 2005, p. 127).

Legitimated regression is acceptable only under the premise that rules and informal social contracts (e.g., fair play, sportsmanship, and norm) are observed (Bredemeier and Shields, 1986a,b; Shields and Bredemeier, 2005, 2008). In general, legitimated regression is not detrimental to everyday moral abilities; it is temporary and is intended to pursue an exceptional purpose (e.g., a goal, such as victory, or fun) as agreed upon by the participants. In other words, self-centered thinking for victory or the act of hitting based on rules is protected by the playful context, rules, and tacit agreement. Thus, such legitimated regression is not an immoral act, but a manifestation of the pursuit of excellence or fun (Shields and Bredemeier, 2008).

However, outside of the rules and norms such behaviors cannot be justified simply because they are “pursuing victory” or are “part of play.” Violation of play rules and tacit norms not only undermines the exceptionality of the game, but also makes game behavior a part of the real problem. Cheating can undermine the ethical values of competition in sports and games and, if serious, impair participants’ moral abilities. Competition in sports and games is not just about winning, it also allows participants to test and learn their physical limitations and moral abilities (Serrano-Durá et al., 2020). That is why most games and sports require rules and ethical commitments to prevent unlimited attacks and reveal physical excellence in a determined way. However, cheating is fundamentally at the same time as the basic premise (fair competition) of sports and games that seek the positive value of competition. This is because cheating occurs by blindly prioritizing victory byproducts (honor, money, unlimited violence, etc.) over the value inside the Magic Circle.

Thus, this “illegitimated regression” cannot be used to justify egocentric behavior that violate the rules. In this regard, Shields and Bredemeier (2005, p. 128) reported that acts outside the scope of the game including hurting others and cheating weaken the competitive structure of the game and can be considered examples of illegal regression. As cheating in sports and games corresponds to “illegitimated regression,” it cannot be justified as an act of “pursuing victory.”

### Potential Factors Related to Cheating in Online Games

In sports, it has been reported that cheating undermines fairness, which results in the destruction of the foundation for a competitive structure and leads to destructive competition (Yan and Randell, 2005; Botvich et al., 2010). Cheating could reduce the trust of the community in the competition structure (game companies, sports federations, etc.) and could even trigger de-socialization (Yan and Randell, 2009; De Paoli and Kerr, 2012; Wu and Chen, 2013). These reports imply that cheating could be a toxic act that negates the ethical commitment of fair play to realize fair competition.

Cheating in online games is also regarded as “illegitimated regression” which cannot be justified. Thus, similar to the general negative effect of cheating in sports, cheating in online games is not free from ethical issues. It has been reported that cheating in games degrades the performance of the fair players and adversely affects the reputation of the competition system (Duh and Chen, 2009; Yan and Randell, 2009; Wu and Chen, 2018). Chen and Ong (2018) pointed out that unlike harmless anomalous behavior, cheating in games has malicious intentionality, and the potential profits from misbehavior to achieve one’s core gaming goals are limited to specific players.

From the concerns on the side effects of cheating, there has been much research on the related factors to cheating in sports. Looking at prior research on dishonest behavior in sports and competitive situations, participants’ motivations and psychological qualities have been reported to trigger or inhibit cheating behavior by affecting perceptions and motivations regarding competition and victory (Rigdon and D'Esterre, 2015; Mudrak et al., 2018).

On one hand, users with external motivations (e.g., competitive motivation of advancement) were more likely to show a low level of sportsmanship. This is likely to apply in the online gaming environment. For example, for the younger generation settled into a common leisure culture, high game scores, rank, tier, etc. can be used as a measure for showing off and can also be linked to monetary rewards in the context of campus leagues and e-sports. For example, a study on adolescents’ game culture and cyberbullying pointed out that “high game rank” in the context of game culture can be a potential motivating force to exercise dominance over others or to cause conflict (Ballard and Welch, 2015; McInroy and Mishna, 2017). Thus, gamers who are motivated to earn external rewards, such as honor or points, may be at higher risk of dishonest behavior than participants who want to maintain high self-esteem through mastery or fun of the game. Likewise, psychological qualities, such as aggression, can influence the means of gaming and increase immoral game behavior for the realization of inappropriate motives, such as relieving suppressed serenity (Thacker and Griffiths, 2012; Lee et al., 2019).

On the other hand, users with inherent motivations, such as improved self-esteem through mastery or fun, seem to have a high level of sportsmanship. Ryska (2003) has shown that sports players with high level of self-esteem or mastery are more likely to have a high degree of sportsmanship. Low self-esteem is related to disruptive behavior in sports, such that cheaters are generally known to show lower levels of self-esteem than non-cheaters (Miller et al., 2007; Błachnio, 2019). Thus, this study focuses on competitive motivation, self-esteem, and aggression as primary factors related to cheating in online games.

### Competitive Motivation and Cheating

Competitive motivation is one of the main factors that could encourage cheating. Earlier studies in the academic and sports fields revealed that the competition attribute has a positive relationship with cheating (Whitley, 1998; Taylor et al., 2002; Rigdon and D'Esterre, 2015). For example, higher degrees of competition pressure or motivation can induce individuals to engage in unethical behaviors by stimulating their desire to be ahead of others (Taylor et al., 2002; Rigdon and D'Esterre, 2015). The fierce competition environment is likely to induce immoral behaviors by encouraging individuals’ moral disengagement (Corrion et al., 2009; Hartmann and Vorderer, 2010).

Other studies in this regard also point to the fact that over-emphasizing success and achievement in competition undermines the positive value of the sport and causes dishonest behavior, such as doping (Yesalis and Bahrke, 2000; Petróczi, 2007; Mudrak et al., 2018). According to self-decision theory in particular, the context of sports and games that beat the other party or emphasize external rewards can negatively affect the perception and judgmental ability of participants regarding certain actions by weakening their inherent motivations (Deci and Ryan, 1985; Vallerand and Losier, 1999). If an individual’s inherent motivation is weakened by a hard competitive structure and an obsession with competitive winning, participants are more likely to overestimate external factors, such as wins and monetary rewards, than internal factors, such as fun or mastery. The increase in external motivation can relatively intensify the psychological pressure toward cheating, which can easily achieve purposes, such as victory or reward. In this context, some studies have pointed out that in order to curb dishonest sports behavior, participants need to be motivated in a way that emphasizes fighting well over conquest or victory (Mudrak et al., 2018).

Related to competitive motivation, high levels of competition rewards can also significantly encourage cheating (Preston and Szymanski, 2003). The tendency to cheat can become much stronger if the potential gains from the misbehavior outweigh the risks it poses. In other words, the likelihood of cheating increases when an individual judges that cheating can yield valuable outcomes, such as high scores, high prizes (or virtual items), a good reputation, and respect from one’s peers, which could increase the degree of competitive motivation (Wu and Chen, 2013; Conrads et al., 2014; Kajackaite and Gneezy, 2017).

Motivations to play games have been found to play key roles in games as in-game behaviors or gaming patterns (Yee, 2006a,b; Billieux et al., 2013). Previous studies have confirmed that players with a high competitive motivation are more likely to show unsportsmanlike or antisocial behaviors in games because they are more greatly affected by frustration and anxiety due to competition pressure than those who do not have a high competitive motivation (Eastin, 2007; Gitter et al., 2013; Lee et al., 2019). Therefore, the potential correlation between competitive motivation and cheating can be equally applied to competitive online games. In particular, most online games, such as LoL, adopt a system that allows high-scoring gamers to compete among users, such as providing insignia, icons, and special custom skins or by desensitizing points in the event of a defeat. This competitive structure deepens the psychological pressure of the user to win the competition and results in an effect that encourages competitive motivation.

A high level of competitive motivation may lead to excessive expectation of rewards and obsession with victory and may lower the degree of psychological rejection of cheating. Thus, the following hypothesis is proposed.

*H1*: Competitive motivation increases the degree of cheating in online games.

### Cheating With the Degree of Self-Esteem

Self-esteem refers to the extent to which one perceives others’ affection for oneself, or the extent to which one has positive views about oneself (Brown et al., 2001). High self-esteem encourages one to make moral choices and forbids one to engage in antisocial or dishonest behaviors (Dai et al., 2002; Donnellan et al., 2005). On the other hand, people with low levels of self-esteem are vulnerable to problematic situations due to their lack of confidence in themselves.

Previous studies have shown that cheaters generally have lower levels of self-esteem and self-efficacy than non-cheaters (Miller et al., 2007; Blachnio and Weremko, 2011; Błachnio, 2019). This result implies that people who are more afraid of failure and who lack self-esteem are more likely to use cheating to achieve their goals (Fontaine, 2006), and also suggests that low levels of self-esteem can adversely affect the behaviors that comply with rules and norms.

The impact of self-esteem on cheating is the same in the sports field. Low self-esteem is known to be a major factor in predicting the use of drugs in sports, or in predicting cheating (Petróczi and Aidman, 2008). After a 4 year tracking research on youth athletes, Laure and Binsinger (2007) discovered that drug users have lower levels of self-esteem than non-users. In addition, a person with high self-esteem through self-awareness is likely to easily lose his or her inherent motivations, even in the context of intense competition, and value internal values (Deci and Ryan, 1985; Harackiewicz et al., 1992; Vallerand and Losier, 1999). This indicates that people with low self-esteem could use cheating to recover their self-esteem through victory in a competition, or to avoid failure.

On the other hand, as people with high self-esteem are likely to have experienced achievements through their own efforts, they do not seem to be attracted by the prospect of winning in a competition through dishonest means. This type of people can maintain or enhance their self-esteem in various ways other than by winning in a competition and have an aversion to involvement in dishonest behaviors (Dai et al., 2002; Donnellan et al., 2005). If these facts are considered, a high level of self-esteem is expected to have a negative correlation with cheating in online games. Therefore, in this study, the following hypothesis was tested.

*H2*: Self-esteem is negatively related to cheating in online games.

Self-esteem is also associated with competitive motivation. Competitions in sports or games provide opportunities to inspire players’ self-esteem by providing goals, challenges, and rewards. The rewards of competition, such as status, grades, money, and points, can be driving forces toward self-esteem. Previous studies showed that people with higher self-esteem are more likely to participate in competitions for achievement than those with lower self-esteem (Pepitone et al., 1967; Costea et al., 2010). Likewise, it was reported that a person with a higher competitive orientation has a stronger concept of self and stronger confidence in self-fulfillment than a person with a lower competitive orientation (Swain and Jones, 1992; Findlay and Bowker, 2009). These findings imply that people with high self-esteem are more likely to try to maintain a positive level of self-awareness and to achieve a sense of accomplishment through competition. In this regard, it is assumed that a high level of self-esteem can affect competitive motivation. In this study, the following hypothesis was tested.

*H3*: Self-esteem is positively associated with competitive motivation.

### Aggression Effects on Cheating

Aggression generally refers to the intention to cause harm to others and the corresponding behavior (Ahsan, 2015). Aggression is cited as a major psychological variable that induces antisocial behaviors, including cheating (Davis and Ludvigson, 1995; Ommundsen et al., 2003; Lucidi et al., 2017). People who show higher degrees of aggression are more likely to show aggressive behaviors than those who show lower degrees of aggression. They are highly likely to have low psychological barriers to antisocial behaviors (Lawrence and Hodgkins, 2009; Smith et al., 2011) and to misjudge that inappropriate behaviors are socially acceptable (Crick and Dodge, 1994, 1996).

These arguments are supported by research results that showed that aggression affects misdeeds in the cyber context or antisocial behaviors (Patchin and Hinduja, 2011; Wang et al., 2017). Likewise, many studies have shown that people with higher levels of aggression and competitive motivation are more involved in cheating than those who are less competitive and aggressive (Davis and Ludvigson, 1995; Błachnio, 2019).

In the sports field, previous research reported that people who have antisocial and aggressive tendencies are more likely to engage in cheating (Ommundsen et al., 2003; Lucidi et al., 2017). In the same vein, some online game studies have reported that people with aggressive and sadistic personality traits are more likely to break rules and show unsportsmanlike behaviors (Thacker and Griffiths, 2012). Given these facts, the impact of aggression on cheating is expected to be higher in competitive online games. In this study, the following hypothesis was tested.

*H4*: Aggression increases the degree of cheating.

Aggression could also affect competitive motivation. Competitive characteristics are known to be related to aggressive behavior (Dowsett and Jackson, 2019). Frustration in a competition affects aggression, including anger, and other negative emotions of an individual (Dollard et al., 1939, p. 1–8, 30). Likewise, high aggression could provoke a preference for a competitive context. Users with high aggression may more actively prefer competitive situations to give vent to their hostility and anger, and to satisfy their sadistic needs. For example, Willoughby et al. (2012) reported that in gaming studies, adolescents with high aggression more actively preferred games with competitive characteristics. Another longitudinal study of adolescents reported a close association between competitive gaming and aggression (Adachi and Willoughby, 2013). The study found that adolescents with high aggression were more likely to play competitive video games over time. Similarly, competitive video game use also predicted higher levels of aggression. Thus, in competitive online games, such as LoL, aggressive players are likely to show high degrees of competitive motivation. In this study, the following hypothesis was tested.

*H5*: Aggression leads to a high degree of competitive motivation.

## Materials and Methods

### Data Collection

An online survey of players of the online competition game LoL was conducted, and the results were analyzed. To collect player data, survey participants were recruited from the most popular LoL communities in South Korea, i.e., “League of Legends Inven” (lol.inven.co.kr) and “League of Legends Hungryapp” (leaguelegends.hungryapp.co.kr). The players who applied for participation in the survey were given a link to the survey on the online survey platform and were rewarded with mobile vouchers (around 3 USD). Online recruitment notices included hyperlinks with detailed guidance on participants’ rights and privacy, and contact information from researchers. Recruitment for participation in the survey took place for about a week. To proceed with data collection, we used an online survey platform.^1^

Data from a total of 373 players were collected. 329 player data were finally used in the analysis after excluding missing values. The final data set included 283 males (86.0%) and 46 females (14.0%), with an average age of 24 years (SD=5.36). More specifically, 14 people (4.3%) in their teens, 211 people (64.1%) in their 20s, 91 people (27.6%) in their 30s, and 13 people (3.6%) in their 40s and an average daily gaming time of 2.30 h (SD=82 min). Because of the higher preference of males for the competitive online games than females, there were more male users in LoL than female users. For example, Ratan et al. (2015) reported 93.6% of male users from the collected data of 18,627 LoL users, and Brühlmann et al. (2020) also reported 94.0% from the sample of 750 users.

### Measurement

Psychological variables and competitive motivation were used by modifying the scales used in previous studies. In terms of competitive motivation, three items of advancement (of competitive game skills) were used (Park and Song, 2010). The scale was designed to measure the will to win, the performance improvement, and show off in competitive situations. The survey respondents were asked to indicate if they agreed or disagreed to the following statements: “I play games to show off my game skills to others,” “I want my skills to perform to the best of my ability during the game,” and “I want to win even in a losing game” (1=“strongly disagree” to 5=“strongly agree”). The mean was 2.85 (SD=1.05), the scale was internally consistent (*α*=0.838).

To measure self-esteem, six statements from Rosenberg’s self-esteem (RSES) scale were used (Rosenberg, 1965). In this study, self-esteem was measured based on the items corresponding to positive self-esteem: “I feel that I have a number of good qualities” and “I feel that I’m a person of worth” (*α*=0.864). The response format was from 1 to 5 (1=“strongly disagree” to 5=“strongly agree”), and the mean was 3.54 (SD=0.82).

To measure aggression, eight statements from the short-form Buss-Perry Aggression Questionnaire were used (Diamond et al., 2005). The scale has been verified for reliability and validity through additional validation studies (Diamond and Magaletta, 2006) and is configured to measure the content of physical or verbal aggression, anger, or hostility: “If somebody hits me, I hit back” and “I have trouble controlling my temper.” Responses ranged from 1 (strongly disagree) to 5 (strongly agree), and the mean was 2.49 (SD=0.87). The scale was internally consistent (*α*=0.894).

To measure cheating (in online games), three statements were used as: “I have used or looked for a hack (auto-targeting software) or bug play (glitching play),” “I do not think it is bad to play bugs or hacks,” and “For fun, I think it is okay to use a hack or glitching play” (*α*=0.876). Responses ranged from 1 (strongly disagree) to 5 (strongly agree), and the mean was 1.64 (SD=0.92).

## Results

In this study, we explored the factors relating game cheating in online games. Specifically, the relations among aggression, competitive motivation, and self-esteem with cheating were examined. For the analysis, a structural equation model (SEM) was used to examine multiple dependence relationships between the variables.

Prior to the full-scale data analysis, reliability and validity tests of the measurement variables were carried out. The reliability test results included Cronbach’s alpha, composite reliability, and AVE (See Table 1). The discriminant validity results for the constructs are shown in Table 2. In correlation analysis, cheating was negatively correlated with self-esteem (*r*=−0.193, *p*<0.001) and aggression (*r*=−0.127, *p*<0.001), and cheating was positively correlated with aggression (*r*=0.412, *p*<0.05), competitive motivation (*r*=0.139, *p*<0.05). On the other hand, game time and gender were not significant.

**Table 1:** Reliability and discriminant validity of constructs.

	*M*	SD	*N*	Cronbach’s Alpha	AVE	C.R.
Self-esteem	3.54	0.824	6	0.864	0.4902	0.8874
Aggression	2.49	0.873	8	0.911	0.5123	0.8936
Competitive motivation	2.85	1.055	3	0.876	0.6340	0.8379
Cheating	1.64	0.921	3	0.876	0.6817	0.8647
Gaming time	2.49	1.212	1			

**Table 2:** Correlations and discriminant validity analysis.

	Self-esteem	Aggression	Competitive motivation	Cheating
Self-esteem	**0.4902**			
Aggression	0.2134^**^	**0.5123**		
Competitive motivation	0.0129	0.0718^*^	**0.6340**	
Cheating	0.0497^*^	0.2275^***^	0.0275^*^	**0.6817**

*The square root of the extracted average variance is presented in bold font in the diagonal cells for the corresponding construct*.

* *p*<*0.0.05;*

** *p*<*0.001;*

*** *p*<*0.001*.

**Figure 1:**
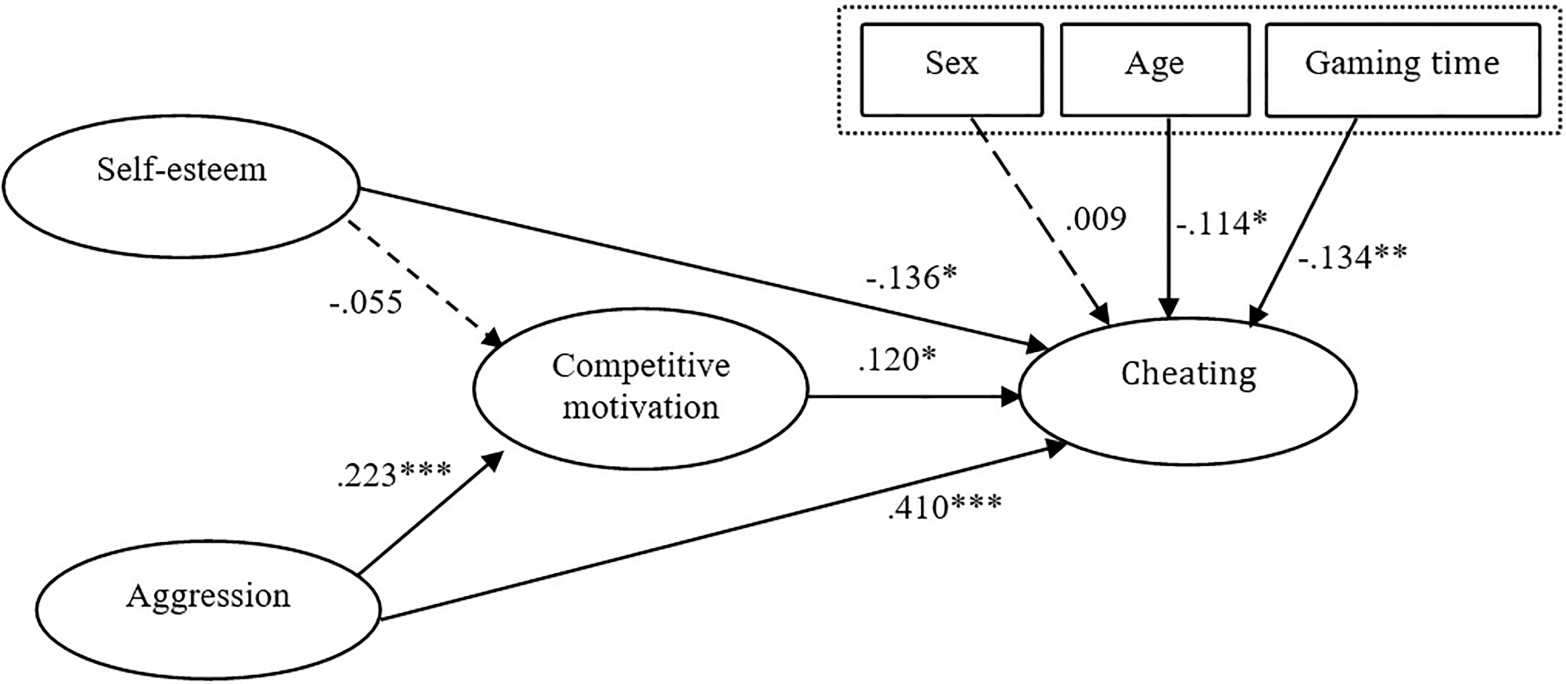
Results of structural equation model. *p<0.05; ^**^p<0.01; and ***p<0.001.

All the scores were found to be valid for the model test (0.8 for composite reliability and 0.5 for AVE; Chin, 1998). For the analysis of the structural equation model, Amos 22.0 was used. The model (See Figure 1) yielded valid indices: CMIN/DF=2.299, IFI=0.944, TLI=0.933, CFI=0.943, and RMSEA=0.063.

The analysis results showed that competitive motivation was positively related to cheating (*β*=0.120, *p*<0.05), and both self-esteem and aggression were significantly associated with cheating. Specifically, aggression showed a strong positive correlation to the level of cheating (*β*=0.410, *p*<0.001), and self-esteem showed a negative correlation to cheating (*β*=−0.136, *p*<0.05). Aggression showed a significant relationship to competitive motivation (*β*=0.223, *p*<0.001). However, there was no significant relationship between self-esteem and competitive motivation (*β*=−0.055, n.s.). Finally, age and gaming time were found to have negative associations with cheating (*β*=−0.114, *p*<0.05 and *β*=−0.134, *p*<0.05, respectively). Table 3 shows the results of hypotheses tests.

**Table 3:** Hypothesis test results.

	*B*	*β*	C.R.	Results
H1. Competitive motivation → Cheating (+)	0.099^*^	0.120	2.194	**Accepted**
H2. Self-esteem → Cheating (−)	−0.155^*^	−0.136	−2.462	**Accepted**
H3. Self-esteem → Competitive motivation (+)	−0.076	−0.055	−0.987	Rejected
H4. Aggression → Cheating (+)	0.407^***^	0.410	6.033	**Accepted**
H5. Aggression → Competitive motivation (+)	0.267^***^	0.223	3.741	**Accepted**

* *p*<0.05; ^**^p<*0.01; and*

*** *p*<*0.001*.

## Discussion

This study empirically examined the associations between psychological factors and cheating in competitive online game environments. Players of a highly competitive teamwork game, LoL, in which they are provoked to win the game with various rewards were recruited for a survey. A structural equation model, specifically with aggression, self-esteem, competitive motivation, and cheating, was analyzed.

The results showed that a high degree of competitive motivation relates to the degree of cheating in the competitive online game. This is in line with previous studies that reported the association of a competitive environment to moral deviation (Taylor et al., 2002; Rigdon and D'Esterre, 2015). Such studies pointed out the association between cheating and competitive motivation in relation to competitive pressure or structure (Whitley, 1998; Hartmann and Vorderer, 2010). High levels of competitive pressure may cause anxiety over the idea of defeat or obsessive compulsion toward victory and may lower psychological barriers to cheating by inducing individual moral deviation (Corrion et al., 2009). The results of this study showed that such risk can be further increased in competitive online games wherein competitive motivation is strongly provoked with the rewards for victory in terms of scope, limited character skins, and rank badges.

There was a negative association between self-esteem and cheating. Self-esteem is linked to an empowering positive perception that difficult challenges can be solved shrewdly by the individual (Brown et al., 2001; Brown and Marshall, 2001), while cheating in games refers to winning points through unjustifiable methods or expediency. Thus, in competitive online game environments, it seems that players with high self-esteem dismiss the idea of cheating by adhering to their method of winning through their own skills.

Aggression significantly positively associated to cheating. This result is consistent with the results of previous studies, which suggests that aggression increases the degree of immoral behavior online (Eastin, 2007; Lee et al., 2016; Lucidi et al., 2017). High aggression not only induces a hostile interpretation of context, but also helps the players vent their aggressive tendencies toward the content. This study result suggests that highly aggressive game players seem to regard illegal programs or malicious bugs as attractive options for releasing their negative feelings. With the help of illegal programs, players could control their anger from losing a game in advance.

Along with this, aggression showed a significant relationship with competitive motivation. Competitive online games, such as LoL, are equipped with “competition” and “reward” factors that justify an aggressive context with provocation of competitive motivation, an “anonymity” factor that induces moral desensitization, and a “violence” factor that allows aggression toward others. In this case, people with higher levels of aggression become more easily immersed in games with competitive motivation compared to less aggressive people, and they are more likely to regard cheating as a means to make sadistic play easier.

Interestingly, aggression showed a much stronger association with cheating than other variables, such as competitive motivation. This result implies that cheating could be mainly caused by users’ aggressive traits, such as hostility and anger, outside the context of competition. This could be supported by the fact that cheating in competitive games is often manifested in extremely violent ways, such as inappropriate attacks on other users or incapacitating opponents (Consalvo, 2009, p. 119–126; Yan and Randell, 2009). Furthermore, considering that competitive online games strongly provoke users’ competitive motivation through situational cues (i.e., competition and reward), the result indicates that cheating could be driven primarily by aggressive traits (e.g., anger and hostility) rather than competitive situational cues. Future studies could compare the effects of situational cues on cheating with those of users’ aggressive traits in such competitive environments.

Notably, gaming time was found to negatively related cheating. This result needs to be interpreted in consideration of the cheating characteristics. Cheating in games provides an unusual gain for a particular player, thereby making it easy to advance further into the game with less effort and time. Specifically, cheating players who receive “abnormal extra points” can easily get rewards, such as rare items, or maintain high scores because they can win much more easily and more often than the average player. In this context, cheating is essentially a behavior that saves time and effort invested in games by violating fair game play. Furthermore, cheating is likely to be seen as an attractive option for players who want to achieve a high rank or get rare rewards but do not have time to do so fairly.

Another explanation could be that such results may be due to the influence of game community norms and game culture. For example, users belonging to a gaming community with hostile norms to cheating may accept the norms of fair competition and have a negative attitude toward cheating. On the other hand, casual game users may be less affected by the norms of honorable and fair competition, and as a result, psychological repulsion toward cheating may be relatively low.

Different from our expectation, however, self-esteem did not show any significant relationship with the degree of competitive motivation. It was expected that players with a high self-esteem would well maintain a positive self-awareness and achieve a sense of accomplishment. Thus, through strong rewards from the competition, self-esteem was supposed to increase competitive motivation in the competitive environment. It seems that the exceptionally highly competitive and rewarding environment of LoL motivated its players to compete without regard to self-esteem. Future studies could compare this result with those of other games that have the level of competition.

This study suggests several implications. It empirically uncovered the meaning of cheating in competitive online games by exploring the association of cheating with psychological and gaming factors. Cheating not only promotes the de-socialization of game players by breaking down the consensus and trust of the game community in fair competition, but also adversely affects game brands by hindering normal gaming operations. To ensure the sustainability of competitive online games, the factors that affect cheating must be disclosed and their occurrence must be minimized.

From a practical perspective, the results of this study on self-esteem may be used in game design to prevent and curb cheating. For example, people with low self-esteem are relatively more susceptible to repetitive failures and can more easily tolerate cheating than people with high self-esteem. Game companies may curb cheating by providing various devices to boost the self-esteem of the players, and by introducing various indicators of the players’ achievements that they can monitor themselves. This is likely to help reduce cheating by strengthening and revealing players’ ethical behavior indicators, and by providing adequate compensation.

In addition, the study suggests that excessive competitive motivation toward victory could result in immoral gaming behaviors. Game companies and game scientists need to actively discover and educate players on the ethical implications and values of “competition” in competitive online games. For instance, it was reported that the moral maturity of the sports community and the settlement of the mastery climate could weaken the performance climate that induces ego-oriented behaviors, and encourage correct achievement ethics (Shields and Bredemeier, 2008). This implies that the work of rediscovering the ethics and value of competitive gaming could effectively help prevent players from cheating in games.

This study had some limitations. First, this was a cross-sectional study in which the causalities between cheating and influencing factors could not be determined. For a more accurate assessment of cheating, subsequent verification through a longitudinal study is deemed necessary. Second, the data used in this study were collected from members of the LoL online community. The results of this study on cheating have limited applicability to other game genres. Future studies could replicate this study in a longitudinal setting with various game genres.

## Data Availability Statement

The raw data supporting the conclusions of this article will be made available by the authors, without undue reservation.

## Ethics Statement

The studies involving human participants were reviewed and approved by Konkuk University IRB. The patients/participants provided their written informed consent to participate in this study.

## Author Contributions

EJ supervised the research and revised the manuscript. SL performed the literature review, data processing, and discussion section. DL and GK performed the data analysis and results section. All authors have read and agreed to the published version of the manuscript.

## Funding

This research was supported by the Konkuk University in 2020.

## Conflict of Interest

The authors declare that the research was conducted in the absence of any commercial or financial relationships that could be construed as a potential conflict of interest.

## Publisher’s Note

All claims expressed in this article are solely those of the authors and do not necessarily represent those of their affiliated organizations, or those of the publisher, the editors and the reviewers. Any product that may be evaluated in this article, or claim that may be made by its manufacturer, is not guaranteed or endorsed by the publisher.

## Footnotes

^1^ https://www.surveymonkey.com
